# Nephroprotective Effect of Embryonic Stem Cells Reducing Lipid Peroxidation in Kidney Injury Induced by Cisplatin

**DOI:** 10.1155/2019/5420624

**Published:** 2019-03-14

**Authors:** Monica Maribel Mata-Miranda, Carlos Eduardo Bernal-Barquero, Adriana Martinez-Cuazitl, Carla Ivonne Guerrero-Robles, Virginia Sanchez-Monroy, Marlon Rojas-Lopez, Gustavo Jesus Vazquez-Zapien

**Affiliations:** ^1^Escuela Militar de Medicina, Centro Militar de Ciencias de la Salud, Secretaria de la Defensa Nacional, Ciudad de México 11200, Mexico; ^2^Facultad de Estudios Superiores de Cuautitlán, Universidad Nacional Autónoma de México, Estado de México 54740, Mexico; ^3^Escuela Nacional de Medicina y Homeopatía, Instituto Politécnico Nacional, Ciudad de México 07320, Mexico; ^4^Centro de Investigacion en Biotecnologia Aplicada, Instituto Politécnico Nacional, Tlaxcala 90700, Mexico

## Abstract

**Introduction:**

The acute kidney injury (AKI) is characterized by a sudden glomerular filtration reduction. Renal or intrinsic causes of AKI include nephrotoxicity induced by exogenous agents like cisplatin, which causes oxidative stress altering the biochemical process and leading to apoptosis. Therefore, this research is aimed at analyzing the embryonic stem cells (ESC) nephroprotective effect in AKI induced by cisplatin, employing genetic, phenotypic, and microspectroscopic techniques.

**Methods:**

Thirty mice were randomly divided into three groups (*n* = 10): the healthy, isotonic salt solution (ISS), and mouse embryonic stem cells (mESC) groups. The ISS and mESC groups were subjected to AKI using cisplatin; 24 h post-AKI received an intraperitoneal injection of ISS or 1 × 10^6^ mESC, respectively. At days 4 and 8 post-AKI, five mice of each group were sacrificed to analyze the histopathological, genetic (*PDK4* and *HO-1*), protein (p53), and vibrational microspectroscopic changes.

**Results:**

Histopathologically, interstitial nephritis and acute tubular necrosis were observed; however, the mESC group showed a more preserved microarchitecture with high cellularity. Additionally, the PDK4 and HO-1 gene expression only increased in the ISS group on day 4 post-AKI. Likewise, p53 was more immunoexpressed at day 8 post-AKI in the ISS group. About biomolecular analysis by microspectroscopy, bands associated with lipids, proteins, and nucleic acids were evidenced. Besides, ratios related to membrane function (protein/lipid), unsaturated lipid content (olefinic/total lipid, olefinic/total CH_2_, and CH_2_/CH_3_), and lipid peroxidation demonstrated oxidative stress induction and lipid peroxidation increase mainly in the ISS group. Finally, the principal component analysis discriminated against each group; nonetheless, some data of the healthy and mESC groups at day 8 were correlated.

**Conclusions:**

The mESC implant diminishes cisplatin nephrotoxicity, once the protective effect in the reduction of lipid peroxidation was demonstrated, reflecting a functional and histological restoration.

## 1. Introduction

The acute kidney injury (AKI) is characterized by a sudden glomerular filtration reduction, provoking the kidney inability to excrete wastes, such as nitrogen products, losing the homeostasis of fluids and electrolytes. Renal or intrinsic causes of AKI include nephrotoxicity induced by exogenous agents like cisplatin, which causes oxidative stress, being the primary site of injury the proximal tubules of the renal parenchyma. The nephrotoxic AKI alters the biochemical process and leads to apoptosis, culminating in acute tubular necrosis (ATN), characterized morphologically by tubular epithelial cell destruction and clinically by the loss of renal function [1].

Currently, the therapies for AKI involve the use of continuous renal replacement techniques indicated for fluid management, correction of electrolyte and pH problems, and treatments for clinical alterations secondary to uremia [2], but unfortunately, these therapies do not resolve the progressive decrease of renal function.

Although these techniques represent the primary treatment against kidney injury (KI), they are associated with high morbidity and mortality. For this reason, it is necessary to explore new alternative therapies for nephropathic patients, seeking a definitive treatment for KI, which might increase tissue regeneration and renal function [3].

Conceptually, the regenerative medicine is a branch of the medicine associated with therapies that regenerate, repair, or replace tissues or the function of an organ by stimulating and inducing its self-regeneration. Schematically, this discipline includes two therapeutic strategies: one based on tissue engineering and the other focused on the use of living cells or cellular therapies. About cell therapies, grafts or implants of mature cells, progenitor cells, or stem cells (SC) are used in this branch [4].

Embryonic stem cells (ESC) have a high capability for self-renewal and pluripotency, which allow them to give rise more SC and differentiate into the three germ lines and their different cell lineages [5]. These cells are mainly obtained from the internal cell mass of the embryo in the blastocyst stage and are characterized by their ability to retain their proliferative properties in an undifferentiated state for an extended period of *in vitro* culture and also by their differentiation into diverse types of specialized cells [6], such as pancreatic [7, 8], cardiac [9], nervous [10], and renal cells [5, 11]. In this sense, new therapeutic options for kidney regeneration are widely studied and tested in animal models of KI [12–16].

The cis-diaminodichloroplatinum (II) or cisplatin (CDDP) is an antineoplastic drug used in the treatment of many cancers in solid organs [17]. Clinical studies have shown that approximately one-third of patients who use this antineoplastic suffer kidney damage, showing an increase in blood urea nitrogen and creatinine, as well as electrolyte imbalance [18].

One of the cellular targets of the CDDP is the nuclear DNA, and this genetic material interaction is the reason of its complex nephrotoxicity mechanisms, involving multiple molecules and signaling pathways such as an imbalance in the prooxidant/antioxidant mechanism promoting oxidative/nitrosative stress, triggering cytotoxicity. Among the molecules of interest, p53 is considered an essential mediator of cell death induced by the CDDP. In this regard, several studies have demonstrated that the CDDP-induced nephrotoxicity increases the expression of p53 in proximal tubules, highlighting that the expression of this proapoptotic protein is correlated with the progression of KI [19–21].

Another mechanism that contributes to the nephrotoxicity caused by CDDP is the inhibition of the synthesis of mitochondrial energy compounds. Fatty acids are the primary source of energy of the proximal tubules, which are the primary target of injury and progression of kidney disease caused by CDDP [22]. About this, *PDK4* (*pyruvate dehydrogenase kinase*) gene induces the inhibition of PDC (pyruvate dehydrogenase complex) by phosphorylating its catalytic subunits E1. The PDC catalyzes the conversion of pyruvate to acetyl-CoA, which results in the oxidation of carbohydrates and regulates the entry of these into the tricarboxylic acid cycle [23, 24]. It is important to mention that *PDK4* is related with the metabolic dysregulation observed in multiple illnesses such as diabetes type 2 [25, 26], hyperthyroidism [27], cardiomyopathy [28], and KI [22, 29].

On the other hand, CDDP can induce the generation of various reactive oxygen species (ROS) by inactivating the cellular antioxidant system, disrupting the mitochondrial respiratory chain, or interacting with the microsomal cytochrome P450, and it is known that the kidney is especially vulnerable to free radicals, because it is one of the main sites for carrying out oxidative processes. Recently, it has been shown that in high states of oxidative stress, there is an overexpression of the enzyme heme oxygenase-1 (HO-1) in renal tubular cells as a protective response to a diverse range of toxic factors [30, 31].

About *HO-1* gene expression, many authors have reported that *HO-1* gene expression induction may serve as an immediate nephroprotective response during CDDP treatment. As aforementioned, oxidative pathways participate in CDDP nephrotoxicity, and the inhibition of *HO-1* increases the ROS levels; contrary, *HO-1* induction protects significantly against CDDP cytotoxicity. In this sense, deficiency or inhibition worsens renal structure and function, and the overexpression of this gene has been associated with nephroprotection. For instance, nephroprotection by global *HO-1* induction using chemical inducers and transgenic mice that overexpress *HO-1* has been demonstrated in ischemia-reperfusion injury, nephrotoxin-induced kidney injury, acute glomerulonephritis, obstructive nephropathy, and rhabdomyolysis [32, 33].

Considering those mentioned above, therapeutic strategies for KI are needed; in previous works, we have demonstrated the mouse ESC (mESC) differentiation capability [5, 8], as well as their beneficial effects when they are implanted in different animal models [34, 35]. Nevertheless, the spectral analysis by Fourier-transform infrared microspectroscopy (FTIRM) after the implantation of mESC in a murine model of AKI and its correlation with the genetic expression have not been studied to show the nephroprotective effect in the lipid peroxidation. Therefore, this research is aimed at analyzing the mESC nephroprotective effect in AKI induced by CDDP, employing genetic, phenotypic, and spectroscopic techniques.

## 2. Materials and Methods

### 2.1. Animal and Study Groups

This experimental work followed the guidelines of the Norma Oficial Mexicana Guide for the use and care of laboratory animals (NOM-062-ZOO-1999) and the disposal of biological residues (NOM-087-ECOL-1995). The animals were males, NIH strain of 2 months old. They were kept in metabolic cages (Allentown Inc.; EcoFlo Rack) in humidity (50-60%) and constant temperature conditions (21 ± 1°C) with a 12 h light/dark cycle and had free access to food and water at all times.

Thirty adult mice were randomly divided into three groups (*n* = 10): the healthy group, isotonic salt solution (ISS) as the control group, and mESC as the experimental group. The healthy group was employed to obtain the normal values of the genetic and protein expression, as well as the spectra of healthy kidney tissue; this group was not manipulated at all. The ISS and mESC groups were subjected to AKI and subsequently received an intraperitoneal (i.p.) injection according to the group that they belonged at 24 h post-AKI. The control group received an i.p. injection of 500 *μ*l of ISS, whereas the experimental group received an i.p. implant of 1 × 10^6^ mESC resuspended in 500 *μ*l of ISS. At days 4 and 8 post-AKI, five mice of each group were sacrificed. One kidney of each animal was designated for the obtention of histological sections for histopathological analysis, p53 immunodetection, and FTIRM, and another kidney was used for RNA extraction for the genetic analysis. Renal function was checked prior assays through creatinine serum levels.

### 2.2. Acute Kidney Injury Induction

All members of the control and experimental groups were subjected to AKI employing 18 mg/kg of CDDP (Sigma-Aldrich; P3494), which was administered by an i.p. injection. Serum creatinine was analyzed 48 h prior AKI to prove the health state of all members of the ISS and mESC groups. In the same way, the KI state was checked at days 4 and 8 post-AKI.

### 2.3. Embryonic Stem Cell Culture

Mouse ESC (ATCC; SCRC-1011) were seeded at a density of 50,000 cells/cm^2^ on a mouse embryonic fibroblast monolayer, employing mESC basal medium (ATCC; SCRR-2010) supplemented with 15% fetal bovine serum, 0.1 mM 2-mercaptoethanol (Invitrogen; 21985023), and 1,000 U/ml mouse leukemia inhibitory factor (Chemicon; ESG1107). The cells were incubated at 37°C in a humidified 5% CO_2_ and 95% air incubator. When cultured cells reached 70% confluency, mESC were characterized morphologically and phenotypically by optic microscopy and immunofluorescence technique, as previously reported [5, 8, 35], to confirm the pluripotency state of the cells. After that, doses of 1 × 10^6^ mESC were obtained and resuspended in 500 *μ*l of ISS.

### 2.4. Histopathological Analysis

The histopathological analysis was developed at days 4 and 8 post-AKI, sacrificing five mice of each group in each day. As aforementioned, one kidney of each animal was used for this purpose, including the immunocytochemistry and microspectroscopy analysis. For the histopathological and immunocytochemistry analysis, the kidneys were embedded in Tissue-Tek (Sakura; 4583) and frozen; subsequently, three kidney tissue cryosections of 4 *μ*m were obtained from each kidney using a freezing microtome (Ecoshel; ECO-1900). After that, tissue cryosections were fixed in 4% paraformaldehyde for 30 minutes at room temperature and rinsed with phosphate-buffered solution (PBS). Afterward, hematoxylin & eosin staining was performed according to the standard methods. Stained sections were analyzed using a light microscope (Nikon; Eclipse Ti-U) and the software Image-Pro Premier 9.1 (Media Cybernetics).

### 2.5. RT-qPCR Assays

Similar to the histopathological analysis, the genetic examination was developed at days 4 and 8 post-AKI, considering the remaining kidney for this study. Towards this end, total RNA of kidneys mice was isolated using TRIzol reagent (Invitrogen; 15596-018) following the manufacturer's instructions (Invitrogen; 15596-018); after that, cDNA synthesis was performed using the first-strand cDNA synthesis kit (Invitrogen; 12328-040). RT-qPCR was conducted by using the ABI PRISM 7000 Sequence Detection System (Applied Biosystems, USA). At each cycle, accumulation of PCR products was detected by monitoring the increase in fluorescence of the reporter SYBR Green PCR Master Mix (Applied Biosystems; 4309155). Straight away after the amplification, dissociation curves were run and analyzed to ensure the specificity of the PCR product. The relative expression levels were calculated using the CT method, which uses the arithmetic formula 2^−ΔΔCT^. Relative RNA levels of all tested genes were normalized to the *β-actin* housekeeping gene and were expressed as means ± standard deviation (SD). Primers were designed using the Primer BLAST Software (Table 1).

### 2.6. Immunofluorescence Staining

For the immunodetection of p53, the tissue sections were fixed in 4% paraformaldehyde (Sigma; P6148) for 30 minutes, and then the samples were rinsed with PBS twice. Subsequently, fixed tissues were permeabilized with 0.1% Triton X-100 (Sigma; X-100) in PBS at room temperature for 5 minutes, and after that, samples were rinsed with PBS and incubated with blocking protein (Dako; X0909) for 20 minutes to inhibit nonspecific staining. Immunocytochemistry staining was done using mouse primary antibody anti-p53 (1 : 200, Santa Cruz Biotechnology; Pab1801:sc-98); anti-p53 was incubated for 60 minutes at room temperature. Subsequently, samples were washed with PBS twice, and the conjugated secondary antibody Alexa Fluor 647 goat anti-mouse (1 : 200, Abcam; ab150115) was incubated for 45 minutes in darkness. Finally, the samples were washed with PBS and coverslipped with 10% glycerol. Microscopic observations were performed in fluorescence microscopy (Nikon; Eclipse Ti-U). Three sections of each kidney were analyzed.

### 2.7. Fourier-Transform Infrared Microspectroscopy Analysis

To develop the biomolecular analysis trough vibrational spectroscopy, three tissue sections of each kidney were analyzed by FTIRM using an FTIR microscope (Jasco; IRT-5200) coupled to an FTIR spectrometer (Jasco; 6600), measuring glomeruli and the tubular system from the cortex and medulla in triplicate. For which purpose, cryosections of 5 *μ*m were obtained and mounted on a gold-coated microscope slide with a gold layer thickness of 100 nm (Aldrich; 643246-5EA). After that, employing a Cassegrain objective of 16x, kidney tissue was focused and dried at room temperature for about 15 minutes to remove excess water, measuring the spectra until the absorption bands related to water were undetectable. Each spectrum was collected in the mid-infrared range (4000-400 cm^−1^) at a spectral resolution of 4 cm^−1^ with 120 scans.

### 2.8. Spectral Analysis

Spectral analysis was performed in the C-H stretching (3025-2800 cm^−1^) and on the fingerprint (1800-800 cm^−1^) regions using a Jasco Spectra Manager software. FTIR absorbance spectra were normalized using a standard normal variate (SNV) normalization employing the Unscrambler X software (version 10.3, Camo). All the spectra of each analyzed microstructure (glomeruli and tubular system of the cortex and medulla) were averaged according to the group that they belonged. Then, the second derivative was calculated employing the Savitzky-Golay algorithm with fifteen point windows and the second polynomial order using the Unscrambler X. Thereafter, the second derivative spectra were analyzed in terms of deconvoluted absorption bands to determine the individual vibrational modes that contribute to the FTIR signal by using a best-fit peak fitting routine of Origin software (version 6.0, OriginLab Corporation), based on the Levenberg-Marquardt nonlinear least-squares method, obtaining the Lorentzian curves whose intensities were used to calculate the ratios corresponding to protein/lipid, olefinic/total lipid, olefinic/total CH_2_, CH_2_/CH_3_, and lipid peroxidation described in Table 2. Each spectral peak was manually selected to define the starting condition for the best-fit procedure. Finally, the Unscrambler X was used to perform the principal component analysis (PCA) in the lipid spectral region.

### 2.9. Statistical Analysis

All data were performed in triplicate, and all experiments were repeated at least three times. Serum creatinine concentrations, gene expression data, the protein fluorescence intensity, and area ratios were presented as mean ± SD. These data were analyzed using one-way analysis of variance (ANOVA), followed by Tukey's test to determine any significant differences. *p* values of less than 0.05 were considered statistically significant.

## 3. Results

### 3.1. Serum Biochemical Analysis

**Figure 1:**
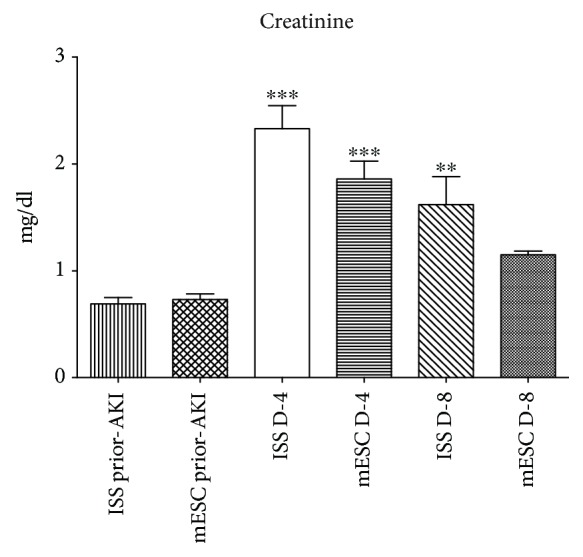
Serum creatinine levels. The graph displays the serum creatinine values of the groups treated with isotonic salt solution (ISS) and mouse embryonic stem cells (mESC) 48 h prior acute kidney injury (AKI), at days 4 and 8 post-AKI. The bars represent the means ± standard deviation (*p* < 0.0001).

As previously mentioned, renal function was checked through creatinine serum levels 48 h prior AKI in the ISS and mESC groups, which exhibited normal values (0.69 ± 0.19 for the ISS group and 0.73 ± 0.17 mg/dl for the mESC group). After that, the creatinine was analyzed on day 4 post-AKI showing a significant increment in the ISS and mESC groups (2.33 ± 0.68 and 1.86 ± 0.53 mg/dl, respectively). Likewise, this metabolite was evaluated at day 8 post-AKI evidencing a decrement (1.62 ± 0.83 for the ISS group and 1.15 ± 0.11 mg/dl for the mESC group) (Figure 1). In the ISS group, a statistical significance was shown at days 4 and 8 post-AKI concerning preinduction values; nevertheless, the mESC group only showed a statistical significance at day 4 post-AKI regarding preinduction values. However, no statistical significance was observed between the ISS and mESC groups.

Once KI was evidenced, histopathological, genetic, phenotypic, and spectroscopic analyses were carried out at days 4 and 8 post-AKI.

### 3.2. Histopathological Description

Five mice of each group were sacrificed at days 4 and 8 post-AKI, and three kidney tissue sections of each kidney were obtained to analyze the tubular system and glomeruli.

**Figure 2:**
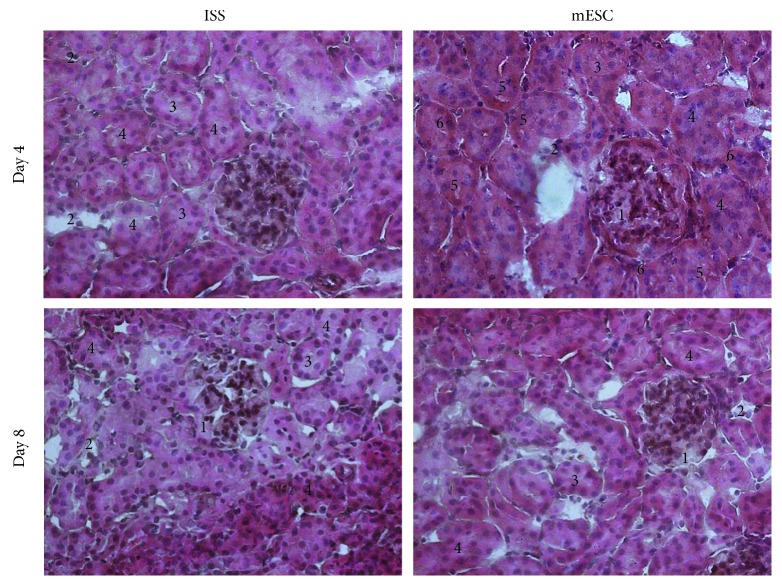
Histopathological analysis. The kidney sections were stained with hematoxylin and eosin, and representative images are shown. The isotonic salt solution (ISS) and the mouse embryonic stem cells (mESC) groups exhibited a segmental focal glomerulosclerosis (1), inflammatory infiltrated (2), tubular dilatation (3), and diffuse denudation of the tubular cells plugging the tubular lumen (4). The mESC group showed tubules less dilated, cytoplasmic vacuoles in the proximal tubular cells (5) and binucleation (6) (*n* = 5 with three biological replicates, 200x).

Both groups (the ISS and mESC groups) exhibited a segmental focal glomerulosclerosis (SFG) (1) and interstitial nephritis characterized by inflammatory infiltrated (2), as well as characteristics related to ATN, such as tubular dilatation (3) and diffuse denudation of the tubular cells plugging the tubular lumen (4). Even though these histological changes were observed in both groups, it is worth to mention that in the mESC group the tubules were less dilated; moreover, cytoplasmic vacuoles in the proximal tubular cells (5) and binucleation (6) were also detected (Figure 2).

### 3.3. Gene Expression

**Figure 3:**
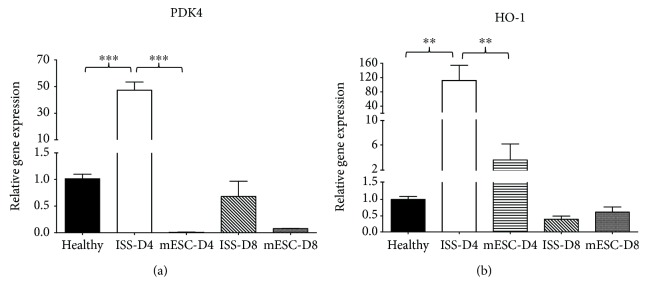
Gene expression analysis. Genes related to metabolic dysregulation (*PDK4*) and oxidative stress and nephroprotective effect (*HO-1*) were analyzed at days 4 and 8 postacute kidney injury. RT-qPCR was performed in triplicate for each sample; bars represent means ± SD. Expression levels were normalized against the housekeeping gene *β*-actin (^∗∗^*p* < 0.001 and ^∗∗∗^*p* < 0.0001).

In this research, genes that encode enzymes involved in the mitochondrial metabolic pathways such as *PDK4* and *HO-1* were studied in the three groups, considering two measurement times for the ISS and mESC groups at days 4 and 8 post-AKI. Changes in the relative expression by RT-qPCR of the genes mentioned above are summarized in Figure 3.

The results showed that *PDK4* gene expression increased up to 47-fold at day 4 in the ISS group with respect to the healthy group; nevertheless, in the mESC group, the expression decreased to 0.01-fold with respect to the healthy group. After that, at day 8 post-AKI, the expression of this gene decreased in the ISS group to 0.7-fold, and in the group treated with mESC, the expression increased to 0.08-fold. It is important to mention that in both groups at day 8 post-AKI, the expression was lower than that in the healthy group (Figure 3(a)).

With respect to *HO-1* gene expression at day 4 post-AKI, as observed in *PDK4* gene, the expression of this gene also increased up to 115-fold in the ISS group with respect to the healthy group; in the same way, the expression increased in the mESC group to 4-fold in relation to the healthy group. Finally, at day 8 post-AKI, the expression decreased in both groups to 0.4 for the ISS group and to 0.6 for the mESC group, almost reaching the expression of the healthy group (Figure 3(b)).

### 3.4. Immunodetection of p53

**Figure 4:**
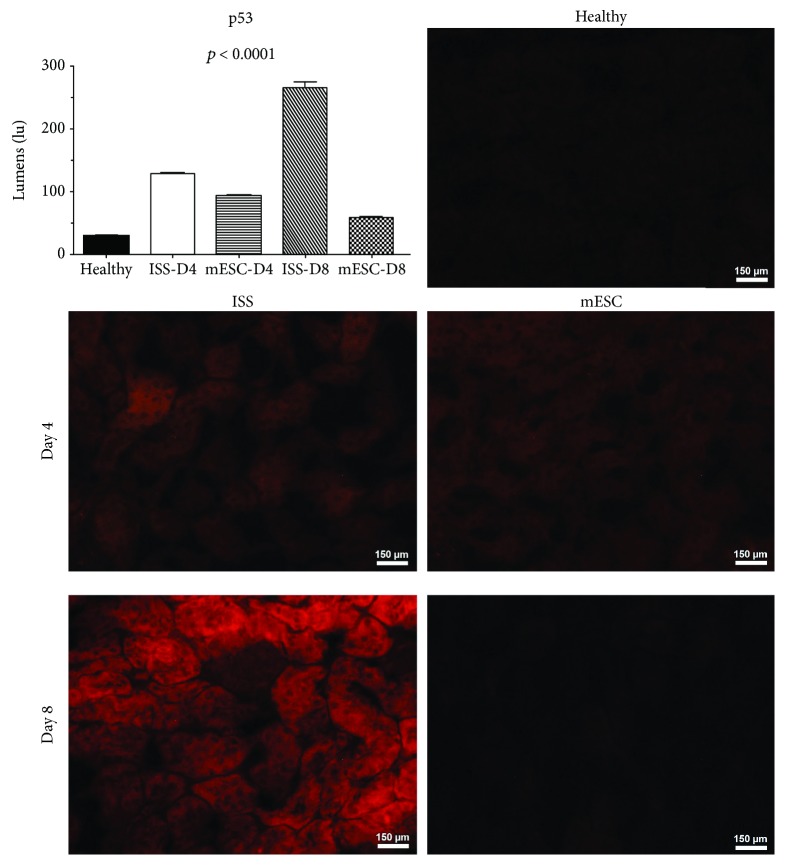
Immunodetection of p53. The graph displays the quantification of fluorescence of the proapoptotic proteins (p53) on kidney tissue; bars represent the means ± SD. *p* represents the value of statistical significance in protein expression between the healthy group and isotonic salt solution (ISS) and mouse embryonic stem cells (mESC) groups. Representative images of immunofluorescence of p53 protein on kidney tissues of mice subjected to acute kidney injury (AKI) treated with ISS or mESC at days 4 and 8 post-AKI (*n* = 5 with three biological replicates, 200x).

Figure 4 shows the immunodetection of the proapoptotic protein p53 in mouse kidney histological samples, specifically in the proximal tubule region. The expression of the protein p53 at day 4 post-AKI was evidenced in the ISS and mESC groups; however, in the group treated with ISS, this expression was higher, increasing significantly at day 8 post-AKI in the ISS groups with respect to the mESC group, highlighting that a statistical significance was observed in all groups concerning the healthy group and between ISS and mESC groups.

### 3.5. Fourier-Transform Infrared Microspectroscopy

**Figure 5:**
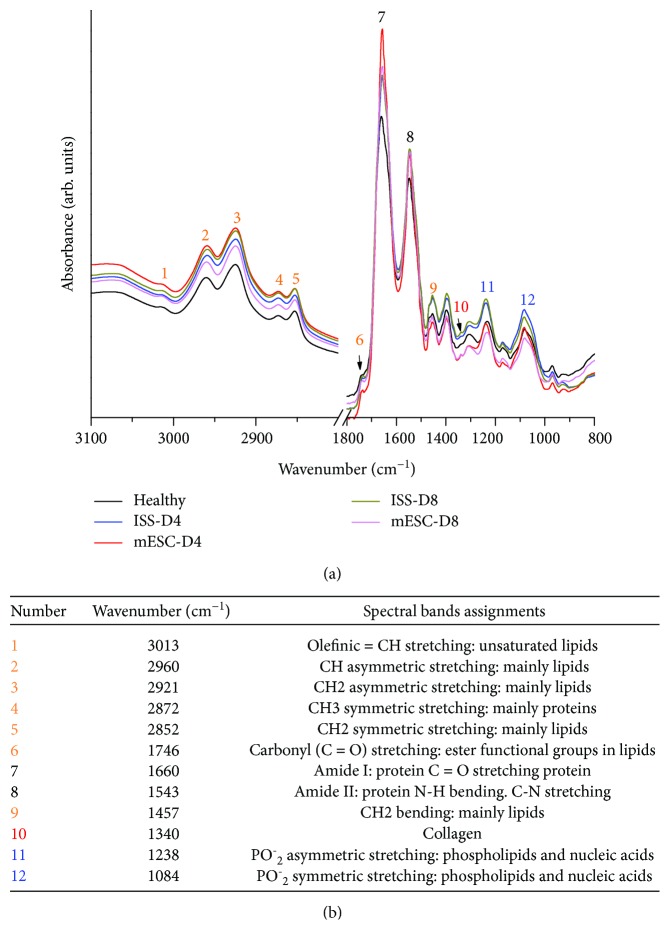
Fourier-transform infrared microspectroscopy (FTIRM) analysis. (a) FTIRM spectra of kidney tissues of healthy mice and mice subjected to acute kidney injury (AKI) treated with isotonic salt solution (ISS) or mouse embryonic stem cells (mESC) at days 4 and 8 post-AKI. Different representative bands associated to biomolecules are evidenced such as lipids, proteins, carbohydrates, and nucleic acids. Each spectrum was collected in the mid-infrared range (4000-400 cm^−1^), depicting the average of glomeruli and tubular system from the renal cortex and medulla of each group (*N* = 5, measured in triplicate). (b) Spectral band assignments.

The averages of the raw and normalized FTIRM spectra of the three groups are shown in Figure 5(a), and the spectral band assignments are summarized in Figure 5(b). Two measurement times for the ISS and mESC groups were considered at days 4 and 8 post-AKI. In the biological fingerprint region (1800-800 cm^−1^), different representative bands associated to biomolecules are evidenced such as lipids, proteins, carbohydrates, and nucleic acids.

Regarding the fingerprint region, in the spectral interval between 1700 and 1500 cm^−1^ related to amide I and II groups of the proteins, an increase in the absorption bands in the AKI groups (ISS and mESC) was shown; at day 4 post-AKI, the mESC group exhibited the highest peak intensity at 1656 cm^−1^ which corresponds to amide I, decreasing at day 8. Nevertheless, in the ISS group, even though an increment in the intensity of this band was observed with respect to the healthy group, it retained almost equal at days 4 and 8 post-AKI. After that, it has shown the following absorption bands at 1457 cm^−1^, 1340 cm^−1^, 1238 cm^−1^, and 1084 cm^−1^, which are related to CH_2_ bending of lipids, collagen, and PO^−^_2_ asymmetric and symmetric stretching of phospholipids and nucleic acids, respectively. These bands show high intensity in the ISS group compared to the healthy group, whereas these bands in the mESC group were lower in intensity with respect to the healthy group (Figure 5).

**Figure 6:**
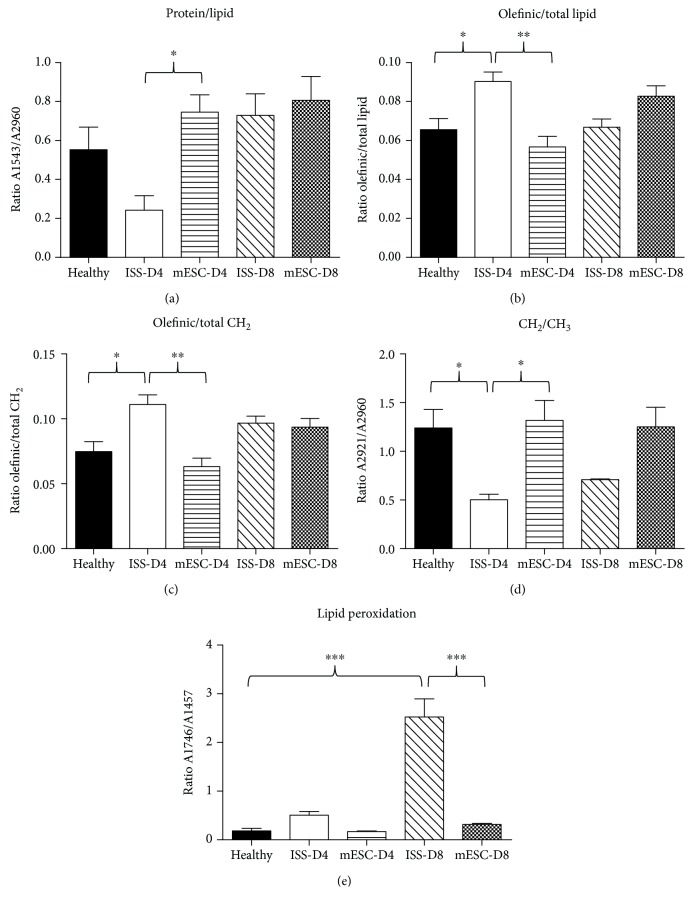
Ratios of structural and compositional alterations obtained from spectroscopic analysis. Ratios related to membrane function, unsaturated lipid content, and lipid peroxidation from Fourier-transform infrared microspectroscopy (FTIRM) spectra of healthy mice and mice subjected to acute kidney injury (AKI) treated with isotonic salt solution (ISS) or mouse embryonic stem cells (mESC) at days 4 and 8 post-AKI. The ratios were as follows: (a) ratio assigned to protein/lipid (1543/2960 cm^−1^), (b) ratio assigned to olefinic/total lipid (3013 cm^−1^/(3013 + 2921 + 2854 + 1746 + 1457 cm^−1^), (c) ratio assigned to olefinic/total CH_2_ (3013 cm^−1^/(2921 + 2854 + 1457 cm^−1^), (d) ratio assigned to CH_2_/CH_3_ (2921/2960 cm^−1^), and (e) ratio assigned to lipid peroxidation (1746/1457 cm^−1^) (^∗^*p* < 0.01, ^∗∗^*p* < 0.001, and ^∗∗∗^*p* < 0.0001).

On the other hand, employing the second derivative spectra, the ratios of protein/lipid, olefinic/total lipid, olefinic/total CH_2_, CH_2_/CH_3_, and lipid peroxidation were calculated (Table 2), in order to determinate structural and compositional alterations, like lipid concentration, acyl chain flexibility, and lipid peroxidation (Figure 6).

Concerning ratio analysis to evaluate the alterations in lipids, Figure 6(a) shows the protein/lipid ratio. Even though we did not find a statistically significant difference between groups, a ratio decrement in the ISS group at day 4 post-AKI with respect to the healthy group was evidenced, while the mESC group presented higher ratio values at days 4 and 8 post-AKI.

Moreover, Figures 6(b) and 6(c) show the ratios of olefinic/total lipid and olefinic/total CH_2_ associated with unsaturated lipid levels. The olefinic/total lipid ratio significantly increased in the ISS group on day 4, decreasing after that on day 8. Nevertheless, in the mESC group, no statistical significance was shown at days 4 and 8 with respect to the healthy group. In the same way, the olefinic/total CH_2_ ratio increased in the ISS group at days 4 and 8 post-AKI, finding a statistically significant difference, whereas the mESC group exhibited a decrement in this ratio at day 4 and a slight increment at day 8 post-AKI, highlighting that no statistical significance was observed in this group.

Regarding chain length of lipids, the CH_2_/CH_3_ ratio depicted in Figure 6(d) displays that this ratio significantly decreased in the ISS group compared to the healthy group; even though in the mESC group a slight increment was evidenced, the ratios were quite similar to the healthy group.

About lipid peroxidation levels (Figure 6(e)), this ratio increased in the ISS group at day 8 post-AKI, finding a statistically significant difference; nonetheless, the mESC group showed almost the same ratios than the healthy group.

**Figure 7:**
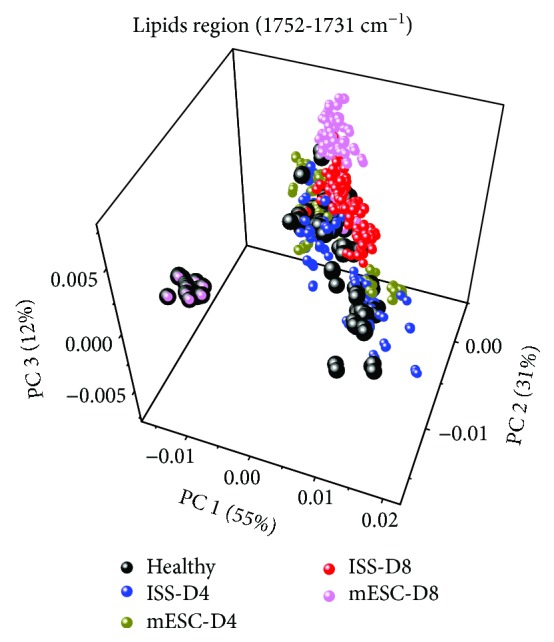
Principal component analysis (PCA) of lipid region (1752-1731 cm^−1^). Score plot (PC1 vs. PC2 vs. PC3) from second derivative Fourier-transform infrared microspectroscopy (FTIRM) spectra of healthy mice and mice subjected to acute kidney injury (AKI) treated with isotonic salt solution (ISS) or mouse embryonic stem cells (mESC) at days 4 and 8 post-AKI.

Finally, to correlate the multiple variables obtained from the second derivative spectra, a PCA was performed in the C=O stretching vibration region of lipids region (1752-1731 cm^−1^). In Figure 7, the first three components (PC1, PC2, and PC3) are depicted explaining the 98% of the total variation of the initial data. This analysis revealed that each studied group is separated in a cluster; nonetheless, some data of the healthy and mESC groups at day 8 are correlated in the PC1 and PC3.

## 4. Discussion

Recent development in cell therapy has demonstrated promising therapeutic effects in KI. Nonetheless, before considering the use of this therapy as a medical option, genetic and biomolecular effects need to be widely studied. For this reason, in this research, we induced AKI in mice employing CDDP, evaluating the mESC implant effects; for which purpose, genetic, phenotypic, and spectroscopic analyses were developed through RT-qPCR, immunocytochemistry, and FTIRM.

Firstly, the KI was confirmed through creatinine serum biochemical analysis. It has been reported that in mice, normal serum creatinine range is 0.630 ± 0.097 mg/dl [36], a value that is quite similar to the one obtained in this research before AKI, confirming that all the animals used in this study were healthy. In the same way, similar to Takai et al. who stated values of serum creatinine of 1.72 ± 0.37 mg/dl 24 h after CDDP (30 mg/kg) administration in mice [36], we reported values of 2.33 ± 0.68 mg/dl in the ISS group and 1.86 ± 0.53 mg/dl in the mESC group at day 4 post-AKI, evidencing a remarkable increase of creatinine in both groups; nevertheless, at day 8 post-AKI, a decrement was observed in both groups (1.62 ± 0.83 for the ISS group and 1.15 ± 0.11 mg/dl for the mESC group), which is related to a better renal function. Although no statistical significance was observed between the ISS and mESC groups, it is important to mention that neither was between the creatinine values obtained in the mESC group prior AKI and at day 8 post-AKI, which could be related to the initial renal function restoration.

Regarding histopathological analysis, the classification of the World Health Organization for tubulointerstitial diseases considers the etiology, clinical, and histological characteristics, stating that the CDDP causes ATN [37]. In this respect, our results showed that in both groups (ISS and mESC) at days 4 and 8 post-AKI, histological characteristics related to toxic ATN were observed, such as inflammatory infiltrate, tubular dilatation, and cell desquamation which plugged the tubular lumen (Figure 2). These results agree with Takai et al. who examined mouse kidneys obtained 72 h after CDDP administration, reporting tubular necrosis, dilatation, and hyaline cast [36]. In the same way, agree with Liu et al. who i.p. injected 20 mg/kg of CDDP in mice, reporting severe pathological changes characterized by the distortion of the overall kidney morphology, mainly dilation of renal tubules [38] and also with Ciarimboli et al. who i.p. injected 15 mg/kg of CDDP, reporting 4 days after AKI signs of renal toxicity such as tubular protein casts and vacuolization of proximal tubular cells [39]. Nevertheless, in the mESC group, although intratubular obstruction and inflammatory infiltrate were also observed, the microarchitecture was more preserved, and the tubules were less dilated; in the same way, in the proximal tubular cells, cytoplasmic vacuoles were detected. About this, it is known that cells with reversible lesions can be microscopically identified by the presence of cloudy swelling or hydropic degeneration, being the result of ion and fluid homeostasis that leads to an increase of intracellular water. Besides, binucleation was also shown, representing a consequence of cell injury and a sort of chromosome hyperplasia which is usually seen in regenerating cells. These results suggest that in the mice that received the mESC treatment, the pathological process of nephrotoxicity was stopped in the early stages, promoting a regeneration process [40].

With respect to RT-qPCR analysis, it is known that the overexpression of *PDK4* leads to the enhancement of fatty acid oxidation (FAO) and decreases the glucose oxidation [41]. Likewise, Li et al. and Oh et al. have reported that PDK4 mRNA and protein levels are markedly increased in the kidneys of mice treated with CDDP [22, 29], which also was seen in this research on day 4 in the ISS group; nevertheless, in the mESC group, this upregulation was not seen. It is important to mention that CDDP induces the upregulation of *PDK4* producing mitochondrial dysfunction, ROS excessive production, and lipid accumulation. Therefore, *PDK4* downregulation could hold therapeutic potential for preventing cisplatin-induced kidney injury [29].

Moreover, as previously mentioned, CDDP can also induce the generation of various ROS that have a high toxic potential to produce diseases such as AKI through the interruption of the mitochondrial respiratory chain, inactivation of the cellular antioxidant system, or the interaction with the microsomal cytochrome P450 [31]. About this, the kidney is especially vulnerable to free radicals, because it is one of the most critical sites for oxidative processes. A significant change in cellular redox may represent a sufficient stimulus for the induction of the expression of genes such as *HO-1* [30] that encodes for a rate-limiting enzyme which catalyzes group heme into carbon monoxide, iron, and bilirubin [42]. Recent studies have reported that in high states of oxidative stress, the *HO-1* expression is induced as a protective response of cells exposed to diverse toxic factors [43]. Our results revealed a significant increase in the expression levels of *HO-1* at day 4 in the ISS group (Figure 3), indicating that this group was subjected to oxidative stress. In this sense, the implantation of mESC reduced the environment of oxidative stress in kidney tissue, which also agrees with the histopathological analysis, once the restoration of the microarchitecture in the mESC group was evidenced.

On the other hand, according to the protein analysis, it is well known that p53 is an essential mediator of cell death induced by CDDP, causing cell cycle arrest and apoptosis, as well as activation of oncogenes and hypoxia. There are a large number of studies related to the expression of this proapoptotic protein and nephrotoxicity by CDDP [18, 20, 21]. Our results showed that in the ISS group, the protein P53 was expressed at day 4 post-AKI increasing its expression considerably at day 8 post-AKI. Nevertheless, in the mESC group, this protein was almost undetectable at days 4 and 8 post-AKI. As previously mentioned, mice treated with mESC showed better renal function and less tissue damage, which correlates with a lower immunodetection of this proapoptotic protein (Figure 4).

Concordant with the genetic and phenotypic analysis, the FTIRM spectral analysis supported the results mentioned above. As previously mentioned, structural information of biomolecules such as lipids, proteins (amide I, amide II, and amide III), and nucleic acids was obtained (Figure 5).

With respect to the amide I region (1700-1600 cm^−1^) related to symmetric stretching vibration of C=O in protein and nucleic acids [43], this band increased in all groups compared with the healthy group; in the ISS group, this increment could be correlated with an augment in protein synthesis, mainly associated with extracellular matrix proteins, related to fibrosis. CDDP inhibits protein synthesis of tubular epithelial cells and triggers proapoptotic molecules associated to inflammatory changes that activate macrophages in response to ROS generation, inducing the expression of inflammatory cytokines such as IL-1, IL-6, TGF-*β*, and RANTES, contributing to the subsequent fibroproliferative process characterized by overexpression and deposition of collagen types I, III, and IV [44–46]. Even though in the mESC group this band was also high, it is possible that this augmentation is not related to an increment in the production of matrix proteins, once it has been reported that changes in proteins and DNA structure could modify amide I band intensity and SC induce glomerular and tubular cell proliferation, increasing cellular survival by secreting proangiogenic and trophic factors, justifying the augmentation of this band with the kidney cell proliferation, which also agree with the histological results once regeneration data such as binucleation were shown. Moreover, Aggarwal et al. and Eirin et al. have reported that SC reduce fibrosis in murine and porcine models of renal stenosis [47, 48].

As previously mentioned, amide I is associated with collagen, reason by which in this research we analyzed specifically the collagen band at 1340 cm^−1^, where an increment in the intensity of this band in the ISS group and a reduction in the mESC group were observed that could be related to fibroproliferative process in the ISS group and a fibroprotective effect in the mESC group, agreeing our results with Liu et al. who reported an increase in the band at 1338 cm^−1^ in liver early fibrosis [49], although it is important to mention that Liu used synchrotron infrared microspectroscopy to characterize the liver fibrosis. Besides, these results also agree with our histopathological results, once glomerulosclerosis was also histologically evidenced.

Additionally, information about the concentration of the biomolecules can be determined from the intensity and the area spectral bands; moreover, the ratios of the band areas provide information about metabolic changes that could correlate to the structure-function relationship [43, 50–52]. In this sense, we analyzed changes in lipid dynamics, such as lipid concentration, acyl chain flexibility, and lipid peroxidation, due to it is reported that biomolecular changes such as lipid alteration, lipid fluidity, protein lipid composition, and the relation between unsaturated/saturated lipids show structure-function relationship, which is related to physiological disorders. Moreover, the band ratios have been used to analyze changes in the cell cycle as well as changes in different metabolic states. In this research, variations in the protein/lipid ratio were observed, which is concordant with the p53 protein expression, results that also agree with those obtained by Yang et al. who reported changes in this ratio due to metabolic changes induced by radiation regulated by p53 [43].

In this regard, as previously mentioned, protein/lipid ratio is related to membrane function. We found a marked decrement of this ratio in the ISS group, especially at day 4 post-AKI (Figure 5(a)), suggesting an increase in lipid content in comparison to protein. Different possible mechanisms could explain these alterations: the oxidative stress induction, mitochondrial dysfunction, and DNA damage altering lipid metabolism and protein content, all those mentioned above induced by CDDP in the proximal tubular cells [53, 54]. CDDP also reduces peroxisomal and mitochondrial FAO leading to the accumulation of toxic fatty acid amphiphiles. This reduction occurs due to the DNA-binding inhibition with the peroxisome proliferation-activated receptor-*α* (PPAR-*α*), decreasing target genes related to FAO and peroxisome proliferator-activated receptor gamma coactivator 1 (PGC-1) formation [22, 55]. The increase in lipid content is also related to *PDK4* gene overexpression in the ISS group at day 4 post-AKI, and according to Li et al., CDDP induces *PDK4* overexpression [22]. In this sense, our results suggest that mESC treatment modulates the protein/lipid ratio and downregulates *PDK4* expression, protecting against CDDP nephrotoxicity effects.

Some clinical and experimental studies suggest that there is a relationship between the progression of kidney damage and alterations in lipid metabolism, mainly related to lipoproteins and triglycerides [56, 57]. The above discussion due to the loss of urinary proteins stimulates a greater LDL synthesis [58], and LDL increase in serum (parameter of nephrotic syndrome) may be attributed to the scarce expression of the LDL receptor (LDLR). In fact, some studies state that patients with nephrotic syndrome exhibit an acquired deficiency of LDLR [59].

Besides, lipid-rich environments are more sensitive to free radical damage and oxidation products [60]. Our results suggest that CDDP increased lipid content showing spectroscopic changes, specifically in the ratios of olefinic (unsaturated lipids)/total lipid and olefinic/CH_2_, which are directly related to variation in lipid metabolism. We found an increase in these ratios in the ISS group at day 4 post-AKI, suggesting an unsaturated lipid increment (Figures 6(b) and 6(c)). Indeed, these ratios have been studied as markers of unsaturated lipid content, highlighting that membrane phospholipids, specifically in the unsaturated lipids, are sensitive targets of free radical, especially under pathological conditions with elevated lipid peroxidation [52]. Additionally, as previously mentioned, CDDP induces free radical production that can interact with membrane lipids increasing lipid peroxidation [61].

All those as mentioned early indicates that one of the nephrotoxic effects induced by CDDP involves lipid peroxidation. About this, Ognjanović et al. reported an increase in lipid oxidation activity related to KI; in our results, we found that CH_2_/CH_3_ ratio decreased in the ISS group, and the ratio A1746/A1457 related to lipid peroxidation increased significantly in this group at day 8 (Figures 6(d) and 6(e)). These ratio changes indicate an increment in lipid saturation, corroborating the lipid oxidation as well as the accumulation of long chain length lipids reported by Yan et al. and Genç et al. [43, 51]. Furthermore, these events are related to apoptosis that also is associated with different membrane changes, such as phosphatidylserine exposure, membrane blebbing, and vesicle formation. This apoptotic process also agrees with the histopathological changes and p53 protein overexpression observed in the ISS group. Our results also agree with Yan et al. who demonstrated that the radiation of HCT116 cells induces lipid oxidation, showed by an A1746/A1457 ratio increment. On the other hand, Shiraishi et al. have reported that *HO-1* overexpression could be related to a reduction of kidney damage, due to *HO-1* degrades heme moiety, acting as scavenging peroxy radical and inhibiting lipid peroxidation [30, 62], which could explain the low lipid peroxidation ratio in the ISS group at day 4 post-AKI, and an augment of this ratio in this group at day 8 post-AKI, which is also related with *HO-1* gene overexpression in the ISS group at day 4 post-AKI. In the same way, the decreased lipid metabolism observed in the mESC group could be related to the low *PDK4* expression, which induced pyruvate dehydrogenase kinase activity.

Finally, there are two ways in which SC recognize the site of damage and repair and replace the injured cells: one is a paracrine action mechanism based on the secretion of growth factors and the other is based on their proliferative and differentiation capability [5, 8, 11, 12]. Nevertheless, broader researches are needed in this field, to establish which of the two mechanisms contributes most significantly to the restoration of the damaged tissue and function.

## 5. Conclusion

According to the obtained results, mESC diminish the CDDP nephrotoxic damage, once the protective effect in the reduction of lipid peroxidation was demonstrated, reflecting a functional and histological restoration. However, it is necessary to study the action and protective mechanisms of the ESC, with the aim to propose new prophylactic strategies in nephrotoxic treatments, expanding the regenerative medicine options.

## Figures and Tables

**Table 1:** Nucleotide sequences of primer pairs used for real-time qPCR.

Gene	Forward 5′-3′	Reverse 5′-3′
*PDK4*	GAGCTGTTCTCCCGCTACAG	CGGTCAGGCAGGATGTCAAT
*HO-1*	CAGAAGAGGCTAAGACCGCC	TCTGACGAAGTGACGCCATC
*β-Actin*	AGAGGGAAATCGTGCGTGAC	AACCGCTCGTTGCCAATAGT

**Table 2:** Ratio assignment obtained from Fourier-transform infrared microspectroscopy (FTIRM).

Ratio name	Chemical description	Spectral assignment
Protein/lipid	Amide II bending N-H and stretching vibration C-N (*δ*NH-*v*C-N)/CH_3_ asymmetric stretching vibration (*v*asCH_3_)	1543 cm^−^^1^/2960 cm^−1^
Olefinic/total lipid	=CH cis stretching vibration (cis*v*C=C-H)/[=CH cis stretching vibration (cis*v*C=C-H)+CH_2_ asymmetric stretching vibration (*v*asCH_2_)+CH_2_ symmetric stretching vibration (*v*sCH_2_)+C=O stretching (*v*C=O)+CH_2_ bending (*δ*CH_2_)]	3013 cm^−1^/(3013 + 2921 + 2854 + 1746 + 1457 cm^−1^)
Olefinic/total CH_2_	=CH cis stretching vibration (cis*v*C=C-H)/[CH_2_ asymmetric stretching vibration (*v*asCH_2_)+CH_2_ symmetric stretching vibration (*v*sCH_2_)+CH_2_ bending (*δ*CH_2_)]	3013 cm^−1^/(2921 + 2854 + 1457 cm^−1^)
CH_2_/CH_3_	CH_2_ asymmetric stretching vibration (*v*asCH_2_)/CH_3_ stretching vibration (*v*asCH_3_)	2921 cm^−^^1^/2960 cm^−1^
Lipid peroxidation	C=O stretching (*v*C=O)/CH_2_ bending (*δ*CH_2_)	1746 cm^−^^1^/1457 cm^−1^

## Data Availability

All the generated data and the analysis developed in this study are included in this article.
